# Land Use Evolution and Land Ecological Security Evaluation Based on AHP-FCE Model: Evidence from China

**DOI:** 10.3390/ijerph182212076

**Published:** 2021-11-17

**Authors:** Yong Zhu, Shihu Zhong, Ying Wang, Muhua Liu

**Affiliations:** 1 School of Business, Hunan University of Science and Technology, Xiangtan 411201, China; 160115030001@mail.hnust.edu.cn; 2 Shanghai National Accounting Institute, Shanghai 201799, China; 3 School of Computer Science and Engineering, Hunan University of Science and Technology, Xiangtan 411201, China; 1050060@hnust.edu.cn; 4 Hunan Engineering Research Center for Intelligent Decision-Making and Big Data on Industrial Development, Hunan University of Science and Technology, Xiangtan 411201, China

**Keywords:** land use evolution, land ecological security, AHP-FCE model, evaluation, China

## Abstract

China experienced rapid urbanization and socioeconomic development at an unusual rate during the past four decades. Against such background, land use evolution and land ecological security have both been affected in a volatile way. Therefore, it is necessary to investigate the land use and the land ecological security in China. However, the traditional assessment approaches have paid more attention to the environmental and economic factors than the sustainable development of ecology, which cannot comprehensively assess the land ecological security. From the perspective of ecological sustainable development, this study identifies 3 main factors and 17 sub-factors. We also construct a model to integrate the FCE approach with the AHP. The results show that from 2004 to 2017, China’s land use structure was unbalanced. The construction land, mining land, and cultivated land increased rapidly, leading to the shrinkage of ecological land. Moreover, the weight of the sustainable development of resources and the environment, economic sustainable development, social sustainable development are 0.3341, 0.3780, and 0.2879, respectively, demonstrating that economic sustainable development is the most important factor affecting land ecological security. Finally, although the value of comprehensive land ecological security in China has been on the rise from 2004 to 2017, it remains at an unsecured level. Moreover, the value of the sustainable development of resources and the environment has been declining since 2011 and is lower than the values of economic sustainable development and social sustainable development. This study demonstrates that more attention should be paid to enhancing land ecological security, especially promoting the sustainable development of resources and the environment.

## 1. Introduction

Land is the fundamental resource and physical condition which humanity depends on for survival and development. Whereas land use and land cover change (LUCC) reflect human activities such as socioeconomic development in the most direct way [1], land ecological security plays a key role in sustainable development for the future of humanity. China is a good research object for studying land ecological security. During the past four decades, China has experienced the rapid urbanization and socioeconomic development at an unusual rate. Against such background, the land use structure in China has witnessed drastic change, giving rise to the contradiction between land ecological security and enormous land demand caused by urban expansion [2,3,4]. Neglectedecological land and its protection, together with the irrational use of land resources, exacerbate ecological imbalance in land. Therefore, as the foundation and key to the sustainable use of land resources [5,6,7], land ecological security maintains the long-term balance in the compound ecosystem of the nature, economy, and society. Dynamic land use change and regional land ecological security and its structure have become vital topics in regional sustainable development [8].

The existing research has mainly focused on the socioeconomic and environmental impacts of land use. Humanity changes the outlook of the world through such ways as expanding agricultural production, increasing urban area, and reducing ecological land, including forest, grassland, and woodland. Land use in different parts of the world may be varied in specific usages. However, the ultimate outcome is similar: The natural environment and ecological security often pay the cost due to the exploitation of natural resources to meet the urgent and insatiable human demand [9,10]. For example, Foley et al. (2005) and Liu et al. (2019) pointed out that land use alters the surface of the earth and disrupts the natural ecosystem, particularly on the land administration, where humans play the dominant role [11,12]. Salvati et al. (2018) indicated that land use change and urban expansion directly affect urban structure and socioeconomic function [7]. Some studies have further unrvaled the impact of land use on climate change, the global ecosystem, the disturbance on global carbon cycle and water resources, and the degradation of biodiversity [13,14]. Studies have echoed the opinion that land use change damages the integrity of ecosystem, which calls for the protection of land and sustainable administration over land use [14,15].

At present, five categories of methods are often employed to the evaluate regional LUCC and the land ecological security: comparative analysis, Geographic Information Systems tools (GIS), regressions, AHP, and the fuzzy comprehensive evaluation (FCE) approach [16,17,18,19,20,21,22,23]. Martínez-Fernández et al. (2019) and Mezősi et al. (2019) compared the land use data of regions in different times, and analyzed their ecological security evolution and trend. They found that regional land use changed dramatically over time, and there was a significant difference in the land ecological security among regions [16,17]. Using Geographic Information Systems tools such as the Corine Land Cover, Castanho et al. (2019) reviewed the land use evolution and characteristics in Poland; examined its development model, future orientation, and impact on economic development; and found that land use plays an essential role in long-term sustainable development [18]. Similarly, Wen et al. (2021) employed a geographic detector to assess the spatiotemporal patterns of land ecological security at both the administrative district scale and grid scale in Chaohu Lake Basin [19]. Feng et al. (2018) used the GIS and generalized additive model to assess the land ecological security in Ningbo city on the southeast coast of China [20]. In addition, Yang et al. (2019) applied the stepwise regression and geographically weighted regression (GWR) to quantify the effects of land use change and urban expansion intensity on landscape patterns [21]. They found that land uses have different contributions to the changes of landscape patterns in the downtown area, suburban plain area, and mountainous suburban areas. Although comparative analysis, GIS, and regressions have certain advantages in accuracy, the results are uncertain, with low accuracy and reliability, and are difficult to widely use.

Further, Han et al. (2015) employed the FCE method to evaluate the land ecological security in several Chinese cities [22]. The results indicated that the socioeconomic indicator contributes more to the improvement of land ecological security. Similarly, Cheng (2022) used the PSR framework and FCE method to evaluate the ecological security of land resources in China [23]. The FCE method processes fuzzy evaluation objects through precise digital means and can provide a more scientific, reasonable, and realistic quantitative evaluation of the data with the hidden information presenting fuzziness. In addition, AHP is more often used in solving complex multi-decision problems [24,25]. Liao (2018) combined the fuzzy comprehensive evaluation method and the analytic hierarchy process to explore the environmental conflict risk in Xiangtan of China [26]. Zhang et al. (2020) used the improved group AHP and FCE methods to investigate the ecological environment impact in highway construction activities. They pointed out that the AHP-FCE model has good applicability and popularization value in the ecological environment assessment [27]. Therefore, this study integrates fuzzy comprehensive evaluation with the analytic hierarchy process to evaluate the land ecological security in China. Our study aims to offer theoretical reference and science-based evidence for the sustainable use of land resources and improvement of land ecological security.

This study contributes to the existing literature in the following aspects. First, our study employs a model integrating the FCE approach with the AHP to provide the empirical evaluation on the land use and the land ecological security in China, which could highlight the complementary advantages of different evaluation methods. Previous literature regarding the land ecological security of China has generally focused on qualitative research, such as concept introduction and comparative analysis, while less quantitative research has focused on the use of the GIS, regression, AHP, and FCE methods alone. As a multi-index compound model, the AHP-FCE method combines quantitative weighting and qualitative indicators, which can comprehensively assess the land ecological security.

Second, this study constructs a resources and environment-economic-social sustainable development analytical framework on understanding the comprehensive status of land ecological security. The existing studies have mainly selected indicators such as environmental factors and economic factors as the main indicators of land ecological security, and the resources and social factors have not been fully considered. However, land ecological security is a complex system, which includes not only the status of land resource utilization itself and the impact of land resource utilization on the economic environment, but also the impact of social development on land use and changes in land carrying capacity. Compared with a single-dimensional or less-dimensional assessment of land ecological security, using the resources and environment-economic-social sustainable development analytical framework is more objective and convincing.

Third, this study measures the status and evolution of China’s ecological security at the national level from a macro perspective. Ecological security assessments related to land often only examine a certain province, city, or river basin in China. Few national-level empirical work has been described in the literature to date. China is a centralized country, and land use in different provinces or cities is strongly related. Therefore, analyzing China’s land ecological security issues at the national level will help to internalize the externalities of land use.

## 2. Methodology

### 2.1. Establishing Indicators System

Based on the theory of sustainable development [28,29], combined with the characteristics of statistical data, the practical operation principles of land ecological security assessment, and the practice of existing literature [30,31], this paper constructs the index system of land ecological security evaluation.

From the perspective of ecological sustainable development, the sustainable development of society and the economy should be equivalent to the sustainable capacity of the natural ecological environment. To ensure the development of human society, we should be within the range that the land ecological environment can carry, and we cannot seek development at the cost of destroying the environment. Only by taking sustainable development as the core, research on land ecological security can highlight its value. Then, combined with the definition of land ecological security, we point out that: (1) Land ecological security emphasizes the sustainability of land ecosystem itself (i.e., the sustainable development of resources and the environment). (2) Land ecological security emphasizes the sustainability of the land ecosystem, providing economic value for human development (i.e., economic sustainable development). (3) Land ecological security emphasizes that human social development should be within the carrying range of the land ecosystem (i.e., social sustainable development).

**Figure 1:**
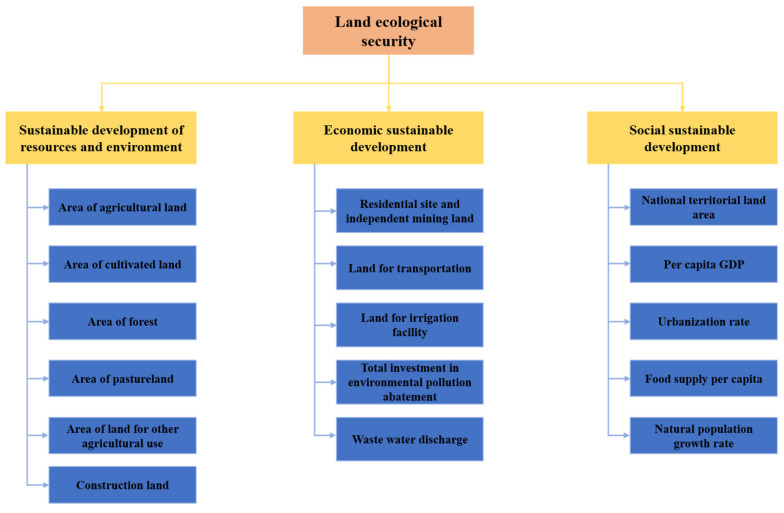
Evaluation framework for land ecological security.

Further, this paper constructs the index system of land ecological security evaluation from three aspects: the sustainable development of resources and the environment, economic sustainable development, and social sustainable development. Specifically, the sustainable development of resources and the environment indicators related to land mainly include the area of agricultural land, cultivated land, orchard, forest, pastureland, and land for other agricultural use and other indicators that can reflect the sustainable state of the ecosystem. Economic sustainable development related to land mainly contain the land use intensity of non-agricultural land, environmental pollution issues, and environmental pollution abatement and other indicators that can reflect the quantity of economic growth and the quality of economic development. Social sustainable development mainly include the national territorial land area, socioeconomic status, urban development status, food supply pressure, population growth rate, and other indicators that can reflect the harmonious development of man and nature. So far, we have built a land ecological security assessment index system with clear levels and clear goals. The specific index system structure is shown in Figure 1.

Data for land use indicators were derived from the China Land and Resources Statistical Yearbook from 2004 to 2017. Indicator data for the total investment in environmental pollution abatement, total waste water discharge, per capita GDP, urbanization rate, food supply per capita, and natural population growth rate were derived from China Statistical Yearbook (from 2004 to 2017). The missing data in the statistical yearbook were complemented by relevant statistical reports.

### 2.2. Establishing the AHP-FCE Approach

**Figure A1:**
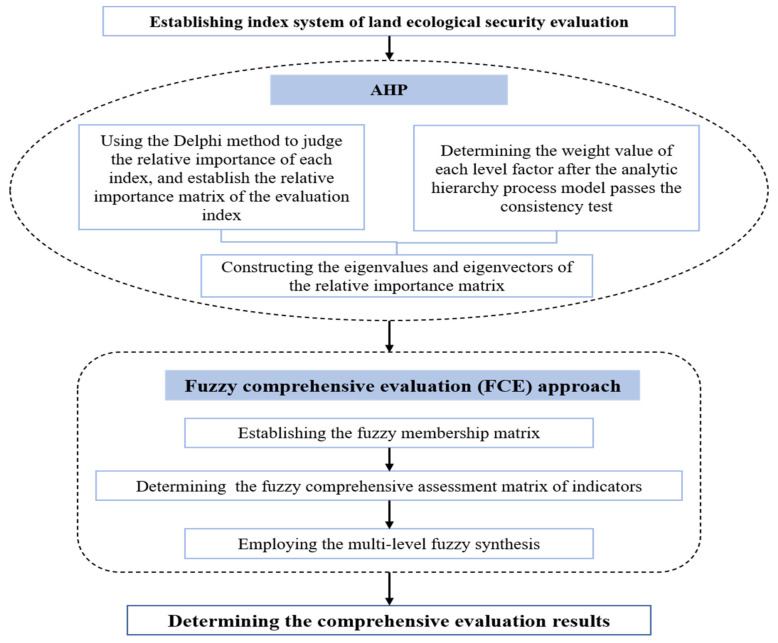
The evaluation process of the AHP-FCE model.

Appendix A (Figure A1) shows the various steps of the analytic hierarchy process-fuzzy comprehensive evaluation (AHP-FCE) approach used to evaluate the land ecological security in China.

#### 2.2.1. Non-Dimensionalizing Assessment Indicator

The evaluation indicators included in this article differ greatly in units. To eliminate the adverse effects caused by oddity sample data, this study used the linear dimensionless method to normalize the values of the assessment indicators [27]. The evaluation indicators in this article can be divided into two categories: the large-value category and small-value category. The large-value indicators are the maximum values that represents the optimal results, such as the area of orchard (S13), area of forest (S14), area of pastureland (S15), land for transportation (S23), land for irrigation facility (S24), total investment in environmental pollution abatement (S25), national territorial land area (S31), per capita GDP (S32), urbanization rate (S33), and food supply per capita (S34). The small-value indicators are the minimum values that represent the optimal results, such as the area of agricultural land (S11), area of cultivated land (S12), area of land for other agricultural use (S16), construction land (S21), residential site and independent mining land (S22), wastewater discharge (S26), and natural population growth rate (S35). Please refer to Appendix B for the specific calculation equation of the large-value category and small-value category.

#### 2.2.2. Establishing the Analytic Hierarchy Process-Fuzzy Comprehensive Evaluation (AHP-FCE) Approach

The AHP-FCE approach was established based on a five-step process [27,32,33].

**Step 1: Establishing the evaluation factors set S**.

Each element in the evaluation factors set S represents an index related to land ecological security, as shown in Equations (1)–(4).

S = {S1, S2, S3}(1)

S1 = {S11, S12, S13, S14, S15, S16}(2)

S2 = {S21, S22, S23, S24, S25, S26}(3)

S3 = {S31, S32, S33, S34, S35}(4)

where S1, S2, S3, respectively, represent sustainable development of resources and the environment, economic sustainable development, and social sustainable development. S11, S12, S13, S14, S15, S16, respectively, represent the area of agricultural land, cultivated land, orchard, forest, pastureland, and land for other agricultural use. S21, S22, S23, S24, S25, S26, respectively, represent construction land, residential site and independent mining land, land for transportation, land for irrigation facility, total investment in environmental pollution abatement, and wastewater discharge. S31, S32, S33, S34, S35, respectively, represent the national territorial land area, per capita GDP, urbanization rate, food supply per capita, and natural population growth rate.

**Step 2: Establishing the assessment set V**.

The assessment set was a collection of various evaluation results. Referring to the practice of Wang et al. (2016) and Wu et al. (2021) [32,34], the assessment set V is described as V = {V1, V2, V3, V4}, in which V1 is safe (S), V2 is relatively safe (RS), V3 is relatively unsafe (RU), and V4 is unsafe (U).

**Step 3: Calculating the single factor membership**.

The triangular membership function method was used to determine the membership degree of evaluation index to realize the fuzzy mapping from the evaluation factors set S to the assessment set V. The {r1, r2, ···, ri} is the set of assessment levels, and the membership function was achieved as Equations (5).

(5) $$f\left(x\right)=\left\{\begin{array}{c} 0,\;x\leq r_{\min}\\ \frac{x-r_{\min}}{r_{i}-r_{\min}},\;r_{\min}<x\leq r_{i}\\ \frac{r_{\max}-x}{r_{\max}-r_{i}},\;r_{i}<x<r_{\max}\\ 0,\;x\geq r_{\max} \end{array}\right.$$

Then, according to the calculation of the single factor membership, the single-factor evaluation matrix was achieved as Equation (6).

(6) $$R=\left[\begin{array}{ccc} r_{11} & \cdots & r_{1m}\\ \vdots & \ddots & \vdots \\ r_{n1} & \cdots & r_{\mathrm{nm}} \end{array}\right]$$

The value of r_ij_ is judged by expert opinion method (Delphi method). In this study, the assessment set V was divided into four levels. Thus, for i=1,2⋯n, there is:

(7) $$r_{i,j}=\frac{V_{i,j}}{\sum_{j=1}^{4}V_{i,j}}$$

To check the consistency of judgment matrix with different order n, based on the practice of Zhang et al. (2018) [35], this study employed the random consistency index (RI) to judge the matrix consistency. The results are shown in Appendix A (Table A1).

**Step 4: Determination of indicator weight**.

The AHP approach was employed to measure the relatively importance of each index factor. This method combines the advantages of qualitative analysis and quantitative analysis. This study used the 1 to 9 comparable scale method to compare the impact degree of the index and establish the judgment matrix. The specific meaning of the 1–9 ratio method is attached in Appendix A (Table A2).

Then, after the consistency test, the weight set of index factor W was achieved as Equation (8).

(8) $$W=\{W1,W2,W3,\cdots ,W17\}\;\mathrm{and}\;\sum_{i=1}^{17}w_{i}=1$$

where W1, W2, W3, W4, W5, W6, W7, W8, W9, W10, W11, W12, W13, W14, W15, W16, and W17 are the weights for S11, S12, S13, S14, S15, S16, S21, S22, S23, S24, S25, S26, S31, S32, S33, S34, S35, respectively.

**Step 5: Multi-level fuzzy comprehensive operation**.

According to the principle of fuzzy transform, this study used the weighted average model to synthesize the single-index evaluation matrix (R) and the weight matrix (W) and obtain the multi-index comprehensive evaluation result-matrix (X), as shown in Equation (9).

(9) $$X_{i}=W_{i}{*R}_{i}$$

## 3. Results

### 3.1. Land Use Type Evolution Analysis

**Figure 2:**
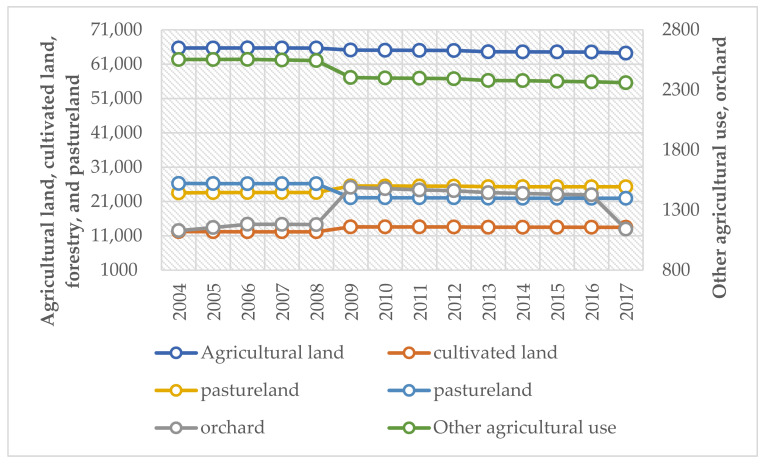
Evolution trend of agricultural and ecological land (2004–2017).

**Figure 3:**
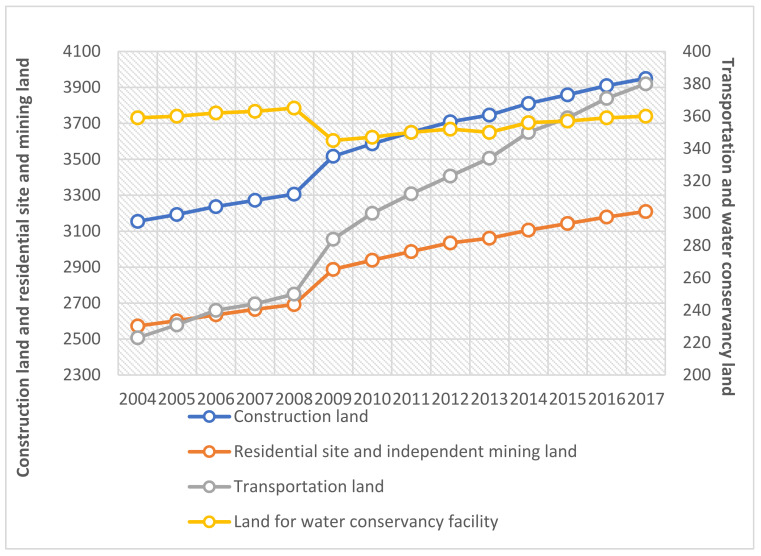
Evolution trend of non-agricultural land (2004–2017).

As exhibited in Figure 2, in terms of the evolution trend of agricultural land and ecological land, pastureland displayed a large decline, followed by land for other agricultural use. Agricultural land declined slightly. In terms of ecological land, pastureland decreased drastically. Forestry increased, while orchards experienced fluctuation. Cultivated land, forestry, and orchards showed an upward trend. Figure 3 displays the evolution of non-agricultural land. Different kinds of non-agricultural land increased in lockstep. The increase of construction land was the largest, followed by that of residential sites and independent mining land.

Overall, since 2007, the proportion of non-agricultural land has continued to increase at an accelerated growth rate while agricultural and ecological land have declined. Construction land, mining land, and cultivated land witnessed the largest increase, with an increase of 25.20%, 24.76%, and 10.18%, respectively. Pastureland and land for other agricultural use experienced a downturn, with a decrease of 16.52% and 7.56%, respectively. The growth rate of non-agricultural land, particularly construction land, far outpaced the decline rate of the proportion of agricultural and ecological land.

### 3.2. Weight Values of Land Ecological Security Evaluation Indicator

As described in Section 2, this study employed the Analytic Hierarchy Process and Fuzzy Comprehensive Evaluation model (AHP-FCE model) to assess the land ecological security in China. According to the steps of the model, we established the sample set of land ecological security evaluation indicators and normalized the evaluation indicators, as shown in Table 1.

Then, we established the fuzzy evaluation matrix based on the normalized value of indicators and constructed the judgment matrix. In addition, we checked the consistency of judgment matrix and calculated the weight. The result show that the consistency coefficient was 0. Therefore, the judgment matrix was considered as consistent. The weights of the indicators were calculated by the AHP method. The specific values are shown in Table 2.

Table 2 shows the weight values of the land ecological security evaluation indicators of China and their ranks. First, for the main factors, the weight of the sustainable development of resources and the environment, economic sustainable development, and social sustainable development was 0.3341, 0.3780, and 0.2879, respectively, demonstrating that economic sustainable development was the most important factor affecting land ecological security. Second, for the sub-factors, the weight of the national territorial land area, residential site and independent mining land, construction land, agricultural land, cultivated land, per capita GDP, land for irrigation facility, and forest was above 0.04. The eight indicators play a leading role in the land ecological security of China. The weight of the orchard, land for transportation, urbanization rate, food supply per capita, natural population growth rate, land for other agricultural use, and pastureland was above 0.02, showcasing a relatively important on land ecological security. The weight of the total investment in environmental pollution abatement and total wastewater discharge was below 0.02, exhibiting little impacts on the land ecological security. This demonstrates that the investment in environmental pollution abatement and wastewater discharge are less important factors affecting land ecological security.

### 3.3. The Multi-Index Comprehensive Evaluation on Land Ecological Security

The comprehensive evaluation value of the land ecological security of China was determined by the value of the sustainable development of resources and the environment, economic sustainable development, and social sustainable development. This study employed Equation (9) to calculate the evaluated value of the sustainable development of resources and the environment, economic sustainable development, and social sustainable development. Based on this, we summed the evaluated value of the main factors to obtain the comprehensive evaluation value of land ecological security from 2004 to 2017. The details are shown in Table 3. We found the land ecological security remained at the unsafe level from 2004 to 2017. Limited by resources and the environment and affected by human activity, land ecological security improvement was obstructed. The land ecological system suffered certain degrees of damage. However, the gradual growth of the comprehensive evaluation value of the land ecological security showed signs of the continuous improvement in land resource protection and in the health of the ecosystem.

**Figure 4:**
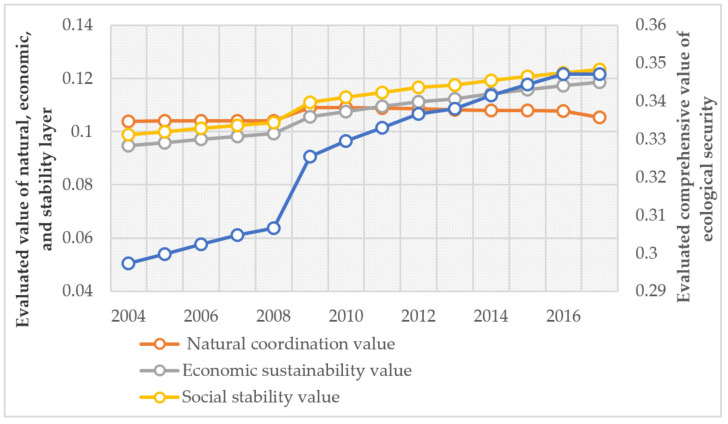
Evolution of land ecological security in China from 2004 to 2017.

To intuitively display the evolution of the land ecological security of China, we drew Figure 4. The results show that, from 2004 to 2017, the comprehensive evaluation value of land ecological security in China experienced little fluctuation and grew gradually. The evaluation value of economic sustainable development and social sustainable development increased to some extent and showed a stable upward trend, while the natural coordination value declined. However, the evaluation value of the sustainable development of resources and the environment showed a downward trend from 2004 to 2017. This may have been caused by the unreasonable use of land by human activities.

In general, the change trend of land ecological security in China is in line with the trend of the sustainable development of resources and the environment, economic sustainable development, and social sustainable development in 2009 and preceding years. However, after 2010, the evaluated value of the sustainable development of resources and the environment began to fall. In addition, its value was continually lower than the value of economic sustainable development and social sustainable development. Affected by this, the rise of the comprehensive evaluation value of land ecological security also slowed. This indicates that sustainable development of resources and environment gradually became the major factor restraining the land ecological security.

## 4. Conclusions and Discussion

China experienced the rapid urbanization and socioeconomic development at an unusual rate during the past four decades. Against such background, land use structure in China has witnessed drastic change, giving rise to the contradiction between land ecological security and enormous land demand caused by urban expansion. Therefore, it is necessary to investigate the land use and the land ecological security in China. Previous studies have used different methods examine the land use and land ecological security in different regions and time dimensions in China, and the conclusions are also quite different. Moreover, these studies have paid more attention to the environmental and economic factors than the sustainable development of ecology, and lacked a systematic analysis framework and composite evaluation system, which cannot comprehensively assess the land ecological security. By constructing the resources and environment-economic-social sustainable development analytical framework, this study identified 3 main factors and 17 sub-factors. We also constructed a model to integrate the FCE approach with the AHP, making the overall weight ratio distribution more reasonable and objective. This study aimed to explore the land use evolution and evaluate the land ecological security in China from 2004 to 2017.

First, we analyzed the evolution characteristics of land use in China. We found that China’s land use structure was unbalanced from 2004 to 2017. The construction land, mining land, and cultivated land increased rapidly, leading to the shrinkage of ecological land. Specifically, construction land, mining land, and cultivated land witnessed the largest increase, with an increase of 25.20%, 24.76%, and 10.18%, respectively. Pastureland and land for other agricultural use experienced a downturn, with a decrease of 16.52% and 7.56%, respectively. The growth rate of non-agricultural land, particularly construction land, far outpaced the decline rate of the proportion of agricultural and ecological land. This may be because, on the one hand, as population continues to grow and urbanization keeps advancing, under limited land resources, construction land and mining land crowd out ecological land. On the other hand, economic development and population growth increase humanity’s demand for cultivated land and energy resources. Driven by the expansion of cultivated land and mining land, economic development continues to increase, leading to the shrinkage of ecological land. In addition, the increase in construction land, cultivated land and mining land will, in turn, promote social and economic development and population urbanization. Under the action of this double-helix mutually reinforcing mechanism, cultivated land, mining land, and construction land have increased rapidly, leading to the further shrinkage of ecological land. Overall, land use is closely related to land ecological security. The decrease in agricultural land and the gradual increase in non-agricultural land, especially the increase in construction land, have had a negative impact on China’s land ecological security.

Second, we analyzed the influencing factors and weight of land ecological security in China. The results showed that the weight of the sustainable development of resources and the environment, economic sustainable development, and social sustainable development was 0.3341, 0.3780, and 0.2879, respectively, demonstrating that economic sustainable development was the most important factor affecting land ecological security. Moreover, the weight of the national territorial land area, residential site and independent mining land, construction land, agricultural land, cultivated land, per capita GDP, land for irrigation facility, and forest was above 0.04, which plays a leading role in land ecological security of China. The eight indicators play a leading role in the land ecological security of China. The weight of the orchard, land for transportation, urbanization rate, food supply per capita, natural population growth rate, land for other agricultural use, and pastureland was above 0.02, showcasing a relatively important on land ecological security. However, the weight of the total investment in environmental pollution abatement and total wastewater discharge was below 0.02, exhibiting little impacts on land ecological security. This shows that a stable land area is the foundation of national land ecological security. In addition, reasonable planning of residential site and independent mining land, construction land, agricultural land, cultivated land, land for irrigation facility, and forest also plays an important role in improving land ecological security.

Finally, we used the AHP-FCE model to obtain the comprehensive evaluation value of land ecological security from 2004 to 2017. We find that, although the value of comprehensive land ecological security in China has been on the rise from 2004 to 2017, it remains at an insecure level. In addition, the change trend of land ecological security in China was in line with the trend of sustainable development of resources and the environment, economic sustainable development, and social sustainable development in 2009 and preceding years. However, after 2010, the evaluated value of the sustainable development of resources and the environment began to fall. In addition, the value was continuously lower than the values of economic sustainable development and social sustainable development. Affected by this, the rise of the comprehensive evaluation value of land ecological security also slowed. It indicates that the sustainable development of resources and the environment gradually became the major factor restraining the land ecological security. In general, although China’s land ecological security has improved, it is still in an unsafe state, especially considering that the level of the sustainable development of resources and the environment has declined rapidly.

The conclusion of this study carries some policy implications. First, efforts should be made to optimize land use structure to reduce the negative impact on land ecological security. It is necessary to increase the area of ecological land and moderately control the construction land, mining land, and cultivated land. Second, we need to continuously improve the level of economic sustainability because it is the most important factor affecting land ecological security. Third, at present, the overall level of land ecological security in China is still low. Therefore, we need to strengthen the protection of land ecological security. More attention should be given to promoting the sustainable development of resources and the environment. However, it is worth noting that, considering the correlation and interdependence between the various subsystems of the land ecological security, improving a single subsystem in the short term may help improve the land ecological security. However, in the long term, this is not sustainable.

There are still several areas for improvement in our study. First, there were limitations in the scope of research. This paper only analyzed the land ecological security of China due to the limitations of data, but it will be very meaningful to extend this research to a wider global scope and conduct comparative analysis. Second, there was insufficiency in measuring the resources and environment-economic-social sustainable development system. We only selected 17 indicators from 3 aspects: the sustainable development of resources and the environment, sustainable development of the economy, and sustainable development of society. It would be of great value to select more corporate- or individual-level indicators from the micro dimensions.

## Figures and Tables

**Table 1:** Normalized value of the index.

Sub-Factors	S11	S12	S13	S14	S15	S16	S21	S22	S23	S24	S25	S26	S31	S32	S33	S34	S35
2004	0.9997	0.8999	0.7582	0.9210	1.0000	0.9996	0.7987	0.8016	0.5868	0.9836	0.1994	0.6561	1.0000	0.2109	0.7136	0.7992	0.9966
2005	0.9998	0.8973	0.7757	0.9237	0.9978	0.9996	0.8084	0.8106	0.6079	0.9863	0.2494	0.7133	1.0000	0.2427	0.7346	0.8192	1.0000
2006	1.0000	0.8950	0.7938	0.9252	0.9970	1.0000	0.8195	0.8209	0.6316	0.9918	0.2680	0.6997	1.0000	0.2827	0.7577	0.8382	0.8964
2007	0.9997	0.8948	0.7931	0.9252	0.9968	0.9980	0.8284	0.8302	0.6421	0.9945	0.3537	0.7573	1.0000	0.3462	0.7842	0.8398	0.8778
2008	0.9995	0.8946	0.7918	0.9250	0.9967	0.9961	0.8370	0.8386	0.6579	1.0000	0.5156	0.7775	1.0000	0.4071	0.8030	0.8807	0.8625
2009	0.9906	1.0000	1.0000	1.0000	0.8405	0.9409	0.8904	0.8997	0.7474	0.9452	0.5492	0.8011	1.0000	0.4422	0.8260	0.8797	0.8268
2010	0.9899	0.9991	0.9926	0.9993	0.8404	0.9393	0.9078	0.9156	0.7895	0.9507	0.7950	0.8394	1.0000	0.5204	0.8536	0.9017	0.8132
2011	0.9892	0.9989	0.9859	0.9985	0.8401	0.9385	0.9241	0.9305	0.8211	0.9589	0.7429	0.8965	1.0000	0.6132	0.8761	0.9381	0.8132
2012	0.9886	0.9983	0.9812	0.9978	0.8399	0.9370	0.9390	0.9455	0.8500	0.9644	0.8619	0.9312	1.0000	0.6735	0.8983	0.9631	0.8404
2013	0.9832	0.9934	0.9704	0.9923	0.8356	0.9311	0.9484	0.9536	0.8789	0.9589	0.9438	0.9458	1.0000	0.7379	0.9181	0.9785	0.8353
2014	0.9826	0.9927	0.9657	0.9916	0.8354	0.9307	0.9648	0.9676	0.9211	0.9753	1.0000	0.9740	1.0000	0.7940	0.9359	0.9819	0.8846
2015	0.9822	0.9922	0.9617	0.9913	0.8352	0.9287	0.9770	0.9791	0.9447	0.9781	0.9197	1.0000	1.0000	0.8451	0.9586	1.0000	0.8421
2016	0.9816	0.9916	0.9584	0.9909	0.8350	0.9268	0.9899	0.9903	0.9763	0.9836	0.9629	0.9671	1.0000	0.9067	0.9800	0.9863	0.9949
2017	0.9769	0.9915	0.7670	0.9905	0.8348	0.9240	1.0000	1.0000	1.0000	0.9863	0.9962	0.9515	1.0000	1.0000	1.0000	0.9808	0.9032

**Table 2:** Weight values of the land ecological security evaluation indicators in China.

Object	Main Factors	Weight	Sub-Factors	Unit	Security Trend	Weight	Weight Rank
Land ecological security evaluation S	Sustainable development of resources and environment S1	0.3341	Area of agricultural land S11	thousand hectares	Negative	0.1176	4
			Area of cultivated land S12	thousand hectares	Negative	0.0994	5
			Area of orchard S13	thousand hectares	Positive	0.0343	9
			Area of forest S14	thousand hectares	Positive	0.0408	8
			Area of pastureland S15	thousand hectares	Positive	0.0205	15
			Area of land for other agricultural use S16	thousand hectares	Negative	0.0207	14
	Economic sustainable development S2	0.3780	Construction land S21	thousand hectares	Negative	0.1185	3
			Residential site and independent mining land S22	thousand hectares	Negative	0.1233	2
			Land for transportation S23	thousand hectares	Positive	0.0304	10
			Land for irrigation facility S24	thousand hectares	Positive	0.041	7
			Total investment in environmental pollution abatement S25	hundred million yuan	Positive	0.0111	16
			Waste water discharge S26	hundred million ton	Negative	0.009	17
	Social sustainable development S3	0.2879	National territorial land area S31	ten thousand hectares	Positive	0.1836	1
			Per capita GDP S32	yuan	Positive	0.0739	6
			Urbanization rate S33	%	Positive	0.0301	11
			Food supply per capita S34	kg/person	Positive	0.023	12
			Natural population growth rate S35	%	Negative	0.0227	13

**Table 3:** The multi-index comprehensive evaluation results of the land ecological security from 2004 to 2017.

Year	Ecological Security Comprehensive Value	Natural Coordination Value	Economic Sustainability Value	Social Stability Value	Security Level	Security Status
2004	0.2973	0.1038	0.0947	0.0988	U	Unsafe
2005	0.2997	0.1040	0.0958	0.0999	U	Unsafe
2006	0.3024	0.1041	0.0971	0.1012	U	Unsafe
2007	0.3047	0.1041	0.0982	0.1024	U	Unsafe
2008	0.3066	0.1040	0.0992	0.1034	U	Unsafe
2009	0.3255	0.1091	0.1055	0.1109	U	Unsafe
2010	0.3295	0.1090	0.1076	0.1129	U	Unsafe
2011	0.333	0.1088	0.1095	0.1147	U	Unsafe
2012	0.3366	0.1087	0.1113	0.1166	U	Unsafe
2013	0.3381	0.1081	0.1124	0.1176	U	Unsafe
2014	0.3416	0.1080	0.1143	0.1193	U	Unsafe
2015	0.3444	0.1079	0.1158	0.1207	U	Unsafe
2016	0.3472	0.1078	0.1173	0.1221	U	Unsafe
2017	0.3472	0.1054	0.1185	0.1233	U	Unsafe

Note: “U” denotes unsafe.

## Data Availability

Publicly available datasets were analyzed in this study. This data can be found here: https://data.stats.gov.cn.

## Appendix A

**Table A1:** Random consistency index (RI) of the judgment matrix.

Matrix Order	1	2	3	4	5	6	7	8	9
RI	0	0	0.52	0.89	1.12	1.26	1.36	1.41	1.46

**Table A2:** The specific meaning of the 1–9 ratio method.

Score	Connotation
1	The two factors are equally important
3	The factor is slightly more important than the other factor
5	The factor is obviously more important than the other factor
7	The factor is significantly more important than the other factor
9	The factor is extremely more important than the other factor
2,4,6,8	Median values of the above adjacent judgments
Reciprocal	If the importance ratio of factor *i* to factor *j* is *b_ij_*, the importance of factor *j* is 1/*b_ij_* as compared to factor *i*.

## Appendix B

**The specific calculation equation of the large-value category and small-value category**.

The dimensionless value of assessment indicator is $r_{i,j}$, $r_{i,j}$∈ [0, 1]. The assessment index set is set to V, and the assessment indicator is $V_{I,j}$∈V. The indicator attribute value is xi, j. The maximum value of indicator is x_max_, and the minimum value is x_min_. The calculations are shown in Equations (A1)–(A3).

(A1) $$r_{i,j}=V_{i,j}\left(x_{i,j}\right)$$

The calculation equation for the judgment where the maximum value represents the optimal result is shown below.

(A2) $$r_{i,j}=\frac{x_{i,j}}{x_{\max}+x_{\min}}$$

The calculation equation for the judgment where the minimum value represents the optimal result is shown as follows.

(A3) $$r_{i,j}=\frac{x_{\max}+x_{\min}-x_{i,j}}{x_{\max}+x_{\min}}$$
